# A Moist Crevice for Word Aversion: In Semantics Not Sounds

**DOI:** 10.1371/journal.pone.0153686

**Published:** 2016-04-27

**Authors:** Paul H. Thibodeau

**Affiliations:** Department of Psychology, Oberlin College, Oberlin, OH, United States of America; Leiden University, NETHERLANDS

## Abstract

Why do people self-report an aversion to words like “moist”? The present studies represent an initial scientific exploration into the phenomenon of word aversion by investigating its prevalence and cause. Results of five experiments indicate that about 10–20% of the population is averse to the word “moist.” This population often speculates that phonological properties of the word are the cause of their displeasure. However, data from the current studies point to semantic features of the word–namely, associations with disgusting bodily functions–as a more prominent source of peoples’ unpleasant experience. “Moist,” for averse participants, was notable for its *valence* and *personal use*, rather than *imagery* or *arousal*–a finding that was confirmed by an experiment designed to induce an aversion to the word. Analyses of individual difference measures suggest that word aversion is more prevalent among younger, more educated, and more neurotic people, and is more commonly reported by females than males.

## Introduction

Many people report that they find words like “moist,” “crevice,” “slacks,” and “luggage” acutely aversive. For instance, *People Magazine* [1] recently coined “moist” the “most cringeworthy word” in American English and invited their “sexiest men alive” to try to make it sound “hot.” One writer, in response, described the video as “…pure sadism. It’s torture, it’s rude, and it’s awful…” and claimed that the only way to overcome the experience was to “go Oedipal and gouge your eyes out” [2]. Indeed, readers who find the word “moist” aversive may experience some unpleasantness in reading this paper.

The current paper addresses foundational questions related to word aversion, focusing on “moist” as a case study since it appears to garner the strongest feelings of aversion among the American public: 1) Approximately what proportion of the population reports an aversion to words like “moist”? 2) Are there individual difference variables that predict who is likely to experience word aversion? 3) Is *aversiveness* a dimension of words that can be measured reliably? And 4) What makes a word aversive in the first place?

Of particular interest is this last question, for which four hypotheses have been proposed. One possibility is that the phonology of certain words is inherently unpleasant. This is an explanation that people with an aversion to the word “moist” sometimes provide: for instance, one participant (from Experiment 1), speculating on their aversion, drew attention to “the ‘oy’ sound juxtaposed to ‘ss’ and ‘tt’. It’s not a word that sounds pleasant. Neither does hoist or foist.” Cognitive scientists have historically viewed sounds in a language as arbitrary with no inherent meaning [3]. However, some have argued that sound symbolism is a natural byproduct of enculturation in a language [4] and cross-cultural studies have found some evidence of sound symbolism beyond onomatopoeia [5–8].

A second possibility, also related to phonology, is that words like “moist” are aversive because speaking them engages facial muscles that correspond to expressions of disgust: a facial feedback hypothesis [9–10]. For instance, one set of studies found that people disliked words with vowels that require speakers to constrict their zygomatic muscles (e.g., as in the German diphthong /yːr/ in *für*)–possibly because such constriction reduces blood flow through the cavernous sinus and raises cerebral temperature [11–12]. The facial feedback hypothesis is controversial, however, and investigations of word aversion may help to shed light on this theory and other embodied views of language and emotion (e.g., [13]).

A third possibility is that the semantic neighborhood of aversive words makes them unpleasant. “Moist” may have become contaminated, a symbol and elicitor of disgust, by virtue of its association with sex or bodily function [14] (e.g., another participant in Experiment 1 said: “it reminds people of sex and vaginas”). On this view, it may be possible to identify a cluster of words in the lexicon as aversive. Such a finding would contribute to a growing literature on the processing of highly valenced and arousing words [15–19] and speak to current debates on the role of culture in the psychology of disgust [20].

A fourth possibility–that aversion to “moist” is merely a fad–would find support in a lack of evidence for the first three hypotheses.

### Overview of Experiments

Five experiments were designed to address specific questions about word aversion. In the first, participants were asked to judge words along a variety of dimensions. Some of the words had similar semantic properties to “moist” (e.g., “damp” and “wet”); some of the words came from lexical categories that commonly elicit disgust (e.g., words that are used in a sexual context like “horny” and “fuck”; and words related to bodily excrement like “phlegm” and “vomit”); others had similar phonological properties to “moist” (e.g., “hoist” and “foist”). If people are averse to “moist” for semantic reasons, they should also find semantically related words and/or words related to disgust unpleasant. If people are averse to “moist” for phonological reasons, they should also find words like “foist” and “hoist” unpleasant.

Because Experiment 1 relied on explicit judgments, which may not accurately track underlying psychological processes, more implicit measures of word aversion were used in Experiments 2 and 3: a free association and surprise recall task, respectively. If participants really do find the word “moist” aversive, then responses in a free association task may help to reveal why [21]. People who find “moist” aversive may be more likely to generate a lexical associate related to disgust–especially if the semantic connotation of “moist” is responsible for the aversion. Moist-averse participants should also be more likely to recall having rated the word in a surprise recall task if it has a stronger emotional valence for them [22].

Experiments 4 and 5 were designed to induce an aversion to “moist” among participants in the sample and thereby test whether an aversion to “moist” is transmitted socially or through a process of conscious deliberation (or both) [20]. People may report an aversion to “moist” because they are conforming to a social norm and/or because, after careful thought, it seems to have phonological properties or semantic associations that make it unpleasant (e.g., as a participant in Experiment 2 explained: “I’m not sure I did [think “moist” was aversive] until other people pointed out that they did. Then it started to bother me as well.”).

Along with investigating differences between people who did and did not report an aversion to moist, we included a contextual manipulation in these experiments. Sometimes “moist” was preceded by items that were designed to prime a sexual or culinary sense of the word (e.g., “pussy” or “cake”); other times it was preceded by unrelated negative or positive words to control for valence (e.g., “retarded” or “paradise”). We expected that priming a positive, culinary, sense of “moist” would make the word seem less aversive and that priming a sexual connotation of the word would make it seem more aversive. We use the results of the context manipulation as a reference point for characterizing the subjective experience of word aversion (e.g., are differences between moist-averse and non-averse participants similar in magnitude to expected differences that result from a context manipulation?).

In each experiment, we asked people to speculate directly on why they found “moist” aversive (if they did), or why they thought other people find the word aversive (if they didn’t). We compare this explicit speculation to the more implicit measures in order to test whether participants’ meta-linguistic awareness aligns with their actual behavior. We also collected several individual difference measures (e.g., of disgust, the Big Five personality dimensions, and demographic variables) to help identify factors of individuals that predict who is likely to experience word aversion.

In addition, in Experiment 5, participants were asked to make a moral judgment about the acceptability of incest between siblings [23]. This scenario has several properties in common with word aversion: both seem to tap into an intuitive, emotionally driven, sense of disgust that people do not seem to describe accurately. The tendency to misattribute the cause of a moral judgment has been coined “moral dumbfounding” [24]. The results of the current experiments suggest an analogous “aversion dumbfounding”: moist-aversion is grounded in semantic associations, although moist-averse participants often point to phonological features of the word as the perceived source of their reaction. Along with highlighting parallels between these psychological phenomena, this measure allows for further investigation of the origin of word aversion. If people are averse to “moist” because the word has sexual connotations, one would expect moist-averse participants to find consensual incest between siblings less acceptable.

Of note, these studies yielded large and complex data sets. The present paper focuses on the specific research questions outlined above and elaborated on below. However, the data have been made available through the Open Science Framework (osf.io/3jwd4) and may provide a foundation for investigating a number of other important research questions.

## Methods

### Ethics Statement

The experiments reported here were done in accordance with the Declaration of Helsinki. Additionally, they followed the ethical requirements of the Oberlin College Institutional Review Board and complied with ethics guidelines set forth by the IRB recommendations; the Oberlin College Institutional Review Board reviewed and approved the protocol for studies presented here. Participants were informed that their data would be treated anonymously and that they could terminate the experiment at any time without providing any reason. We received informed consent from all participants before they participated in an experiment. The first page of the study described the potential risks and benefits of participation. Upon agreeing to these conditions, participants clicked a radio button as an indication of their consent; they were then provided with additional instructions and the experimental materials.

### Participants

Participants in all five experiments were recruited through Amazon’s Mechanical Turk using the same inclusion criteria: participants had to be at least 18 years of age, live in the US, and have a good performance rating (>90% approval rating). Participants were told in advance that they would encounter words that could be upsetting, although no specific words were identified explicitly in the description of the task.

Demographic characteristics of the five samples are shown in Table 1. Participants were not permitted to complete related experiments (e.g., if a participant in Experiment 5 had participated in Experiment 1, their data from Experiment 5 was excluded from analysis). The sample size in Experiment 1 was set to include 100 participants per condition because of the exploratory nature of the work. Sample sizes for Experiments 2–4 were set to be consistent with that of Experiment 1 (100 per condition); the sample size for Experiment 5 was larger (200 per condition) because more attrition was expected.

**Table 1:** Sample Demographics.

	Exp1	Exp2	Exp3	Exp4	Exp5
**Sampled**	400	400	800	400	650
**Analyzed**	400	370	688	377	572
**Gender:** Females	57%	55%	62%	52%	68%
**Age (*mean*)**	35.2	32.6	35.0	35.8	36.8
**English as first language**	97%	98%	97%	98%	98%
**Ethnicity:** White	76%	76%	80%	83%	79%
**Political Ideology** (0 = very liberal; 100 = very conservative)	38.1	37.9	41.0	42.1	41.5

Demographic characteristics of the samples from Experiments 1 through 5.

These five experiments are a nearly exhaustive set of exploratory studies on this topic from our lab. One additional study was conducted (similar to Experiment 3) and is described in the S1 Text. A coding error in this version of the experiment prevented collection of critical information about participants in this sample.

### Materials and Design

The materials and design for the five experiments were similar. The specific tasks that participants completed in each experiment and the order in which they completed them are shown in Table 2; methodological details for each experiment are presented in detail below. In Experiments 1, 4, and 5, participants rated a set of 29 target words along six dimensions. In Experiment 2, participants were exposed to these same words and replied with the first word that came to mind in a free association task. In Experiment 3, participants rated a larger set of words along one of two dimensions (positive or negative connotation); then they were presented with a surprise recall task.

**Table 2:** Experiment Overview.

Exp 1	Exp 2	Exp 3	Exp 4	Exp 5
Ratings of 6 Dimensions	Free Association	Connotation Ratings then Surprise Recall	Averse?	Ratings of 6 Dimensions
Averse?	Averse?	Averse?	Ratings of 6 Dimensions	Averse?
Individual Difference Measures				

Schematic of tasks and design for Experiments 1–5.

In most experiments, participants were asked whether they identified as categorically averse to “moist” at the end of the study. However, in Experiment 4, participants were asked to identify as categorically moist-averse at the beginning–to investigate how the ratings task might influence whether people identified as a categorically “moist” averse (and vice versa).

#### Ratings of 6 Dimensions: Experiments 1, 4, and 5

In Experiments 1, 4, and 5 participants rated 29 words along six target dimensions that have previously been studied in relation to taboo and emotionally valenced words [25]: *personal use*, *familiarity*, *aversiveness*, *valence*, *arousal*, and *imagery* (see Table 3). Of note, Janschewitz’s [25] questions about the *tabooness* and *offensiveness* of words were replaced with a single question about the *aversiveness* of words in the present studies. This change was made because neither *tabooness* nor *offensiveness* seemed to capture the dimension along which words like “moist” are distinctive.

**Table 3:** Definitions of Rated Dimensions.

Dimension	Definition (and scale labels)
**Personal use**	How often do **YOU** use the word in any way–speaking or writing?
	(0 = Never; 50 = Sometimes; 100 = All the time)
**Familiarity**	How often do you **encounter** the word? For example, you may hear it used in a conversation, on the radio, in a movie or on TV, or you may read the word in a magazine, book, or the Internet, etc.
	(0 = Never; 50 = Sometimes; 100 = All the time)
**Aversiveness**	How aversive is this word to **YOU**?
	(0 = Not at all; 50 = Somewhat; 100 = Extremely)
**Valence**	How positive or negative is the word?
	(0 = Strongly negative; 50 = Neutral; 100 = Strongly positive)
**Arousal**	How exciting is the word? Consider how much the word **grabs your attention.**
	(0 = Not at all; 50 = Medium; 100 = Extremely)
**Imagery**	How easily does the word bring an image to mind? When you think of the word, can you picture what it is? If this is easy, the word is high in imagery. For example, a word like "apple" has more imagery than a word like "honor."
	(0 = Doesn’t bring an image to mind; 100 = Brings a vivid image to mind)

Definitions of the six rated dimensions, as presented to participants in the study. Bolded and capitalized words were bolded and capitalized for participants.

Ratings were made on 101-point scales that ranged from -50 to 50; for clarity of presentation these ratings have been shifted up by 50 units so that reported means have positive values between 0 and 100. The rating scales and 15 of the filler words were taken from prior work on taboo and emotionally valenced words [25].

One of the 29 target words was “moist”; the remaining 28 words came from 6 lexical categories, including: 1) five words that were semantically related to “moist” (damp, dank, muggy, sticky, wet); 2) three words that had similar phonological properties to “moist” (foist, hoist, rejoiced); 3) three negatively valenced words relating to bodily function (phlegm, puke, vomit); 4) four words relating to sex (buttfuck, fuck, horny, pussy); 5) four unrelated negative and taboo words (murderer, nigger, retarded, shithead); and 6) nine positively valenced words (brave, cake, delicious, gold, heaven, love, paradise, sunset, sweet).

The words were presented in pseudo-random order. In Experiments 1 and 4, three words, which were designed to anchor participants’ ratings of aversiveness, initiated the survey (murderer, gold, and shithead). These items were followed by two words from one of four categories that were expected to influence the sense of “moist” that participants brought to mind: 1) related and positively valenced (cake, delicious), 2) related and negatively valenced (fuck, pussy), 3) unrelated and positively valenced (paradise, heaven), and 4) unrelated and negatively valenced (nigger, retarded). Words from the two semantically related conditions were designed to prime a sexual or culinary sense of “moist”; the unrelated negative and positive conditions served as a control to the general manipulation of valence. “Moist” was fixed to the sixth position of the questionnaire. The remaining words were presented in random order with one final exception: the word “love” was always the final item that participants’ rated. It was fixed to this position so that participants’ would end the survey having considered a positive item.

There was one difference between the designs of Experiment 1 and Experiment 4. Whereas in Experiment 1 participants were asked to rate the 29 target words before they identified as categorically moist-averse (or not), this judgment was made at the beginning of the study in Experiment 4.

There were two differences between Experiment 1 and Experiment 5. First, in Experiment 5 the word “moist” was positioned at the end of the survey rather than toward the beginning (position 29 rather than 6). Second, the context manipulation in this experiment involved three between-subjects conditions: one third of participants were exposed to a video produced by *People Magazine* [1], in which some of “the sexiest men alive” spoke the word “moist”; one third of participants were exposed to a video of people using the word “moist” to describe the taste of cake; a final group was not shown a video. The second video (cake) was filmed by the researchers and was designed to be similar to the one made by *People*. Both videos included 5 actors who said the word “moist” in quick succession without elaboration (total time: ~30 seconds). Although the actors in the original video occasionally giggled or muttered “gross” or “yuck” quietly after saying “moist,” actors in the control video were instructed not to make such expressions. Instead, they were shown eating a piece of cake and then saying “moist.” Their utterance was often accompanied by a nod or an approving look toward the cake so that it was clear that “moist” was being used to describe a positive experience.

In Experiment 5, participants who watched either video were asked a catch question (e.g., to identify an actor from the video). They were also asked whether the volume on their computer was on. People who responded incorrectly to a catch question or reported they did not hear the audio were excluded from analyses (*n* = 51; 8% excluded).

#### Free Response: Experiment 2

In Experiment 2, participants were presented with the same set of 29 words in the same pseudo-random order as in Experiment 1 (with the same context manipulation induced by words that immediately preceded “moist”). However, instead of rating these words, participants were instructed to reply with the first word that came to mind.

Two independent coders categorized responses to “moist” into five categories, which emerged from reading the range of responses given by participants: *wet*, *yuck*, *sex*, *food*, and *other*. Inter-rater reliability for this coding scheme was high (Cohen’s *Κ* = .835); disagreements were resolved through discussion.

#### Connotation Ratings and Surprise Recall: Experiment 3

Participants in Experiment 3 were asked to rate 64 words – 28 of the 29 items from the previous three experiments as well as 36 additional words (the word “buttfuck” was removed from the set in this experiment because of its similarity to “fuck”)–for their positive or negative connotation. Both sets of ratings were made on a five-point scale: from “Not at all positive” to “Very positive” or from “Not at all negative” to “Very negative.” The scales were carefully designed to focus participants’ attention on the positivity or negativity of the words: for instance, the low end of the positive scale was not anchored with the word “very negative” but instead by a negation of the word “positive.”

The 36 additional words were carefully selected so as not to induce a semantic category effect for the word “moist” [26]. They included mostly positive unrelated (e.g., bride, fruit, happy) or negative unrelated words (e.g., anger, pain, war) taken from prior research [25].

The 64 words were divided into four blocks of 16 items. The order of most of the words varied randomly within their respective blocks; the orders of the blocks were fixed; blocks were presented on separate pages; words from the six categories identified in Experiment 1 (e.g., words related to sex and bodily function) were evenly distributed across blocks. However, the word “moist” was fixed to position 38 (the sixth word in the third block)–a position for which one would expect a low rate of recall [27]. The same three words (murderer, gold, shithead) always initiated the third block and were followed by two words that were either positive or negative and either related or unrelated to “moist” (i.e., the same words used to induce a context effect in Experiment 1).

After rating the 64 words, participants were asked to “write all of the words that you can remember rating on the previous screens.” They were instructed to “do your best to recall the words from memory”; the survey prevented them from going back to previous pages.

#### Moist-aversion: Experiments 1–5

All participants were asked if they found “moist” aversive (yes or no) and to speculate either on “why you find it aversive?” or “why you think other people are averse to it?” (free response). In most experiments, these two questions were asked at the end of the survey; in Experiment 4, this question was asked at the beginning of the survey.

The free response question was coded by two independent raters who categorized the explanations into one of four mutually exclusive and exhaustive categories: those that identified 1) the sound alone; 2) the connotation alone; 3) both the sound and connotation; 4) or neither the sound nor the connotation. Inter-rater reliability was high for this coding scheme (Cohen’s *Κ* was between .7 and .85 in the five experiments); disagreements were resolved through discussion.

#### Individual Difference Measures: Experiment 1–5

Finally, participants in all five studies were asked demographic (i.e., age, gender, educational background, and political ideology) and personality questions: a Ten Item Personality Inventory (TIPI), which is a brief measure of the Big Five personality traits [28]. Three measures of individual difference variables were presented to participants in Experiments 2–5 (but not to participants in Experiment 1): blirtatiousness, which measures the extent to which people respond to others quickly and effusively that has been shown to capture how physiologically aroused a person becomes in response to unpleasant stimuli [29]; a measure of disgust [30]; and a measure of religiosity [31]. The measure of religiosity was included in order to identify the source of a potential link between word aversion and disgust (e.g., a religiously-associated motivation for purity and cleanliness; [32]).

## Results

The results section is organized around specific research questions. The first subsection describes three results that help characterize the phenomenon of word aversion, showing that it can be quite visceral. The second subsection quantifies the prevalence of moist-aversion and identifies characteristics of individuals who report experiencing the phenomenon. The remaining subsections seek to uncover the cause of word aversion by investigating whether people who identify as moist-averse are also relatively sensitive to words that have similar semantic or phonological properties to “moist” and by comparing the lexical profile of “moist” to the profile of disgusting and taboo words.

### What is word aversion?

Ratings data from Experiment 1, free response data from Experiment 2, and recall data from Experiment 3 help to characterize the subjective experience of word aversion. For instance, in Experiment 1, people who reported an aversion to “moist” tended to rate the word as 24.06 units higher on a 101-point scale of *aversiveness*. This between-group difference is comparable to the difference in *aversivness*, in ratings from the full sample of participants, between “nigger” and “phlegm” (23.2 units; two words that were judged to be above the midpoint of the *aversiveness* scale) as well as to the difference between “fuck” and “delicious” (25.8; two words that were judged to be below the midpoint of the *aversiveness* scale).

The context manipulation in this experiment further helps to interpret what it means to be averse to a word. In Experiment 1, the word “moist” was preceded by two words that were either semantically related or not and either negatively or positively valenced. Participants’ rating of the *aversiveness* of “moist” differed as a function of this manipulation, *F*[3, 396] = 2.666, *p* = .048, *η*^*2*^ = .020. People found “moist” more *aversive* when it followed unrelated positive words (*M* = 36.812, 95%CI: [31.304, 42.320]) or sexual words (*M* = 36.188, 95%CI: [30.360, 42.016]) and less *aversive* when it followed food primes (*M* = 31.520, 95%CI: [26.032, 37.008]) or unrelated negative words (*M* = 26.969, 95%CI: [21.591, 32.348]). The largest difference between conditions was 9.84 units, which was the result of a contrast effect between the unrelated negative and unrelated positive conditions [33], and is small compared to the difference between moist-averse and non-averse participants (Cohen’s *d* = .355 compared to .854).

**Fig 1:**
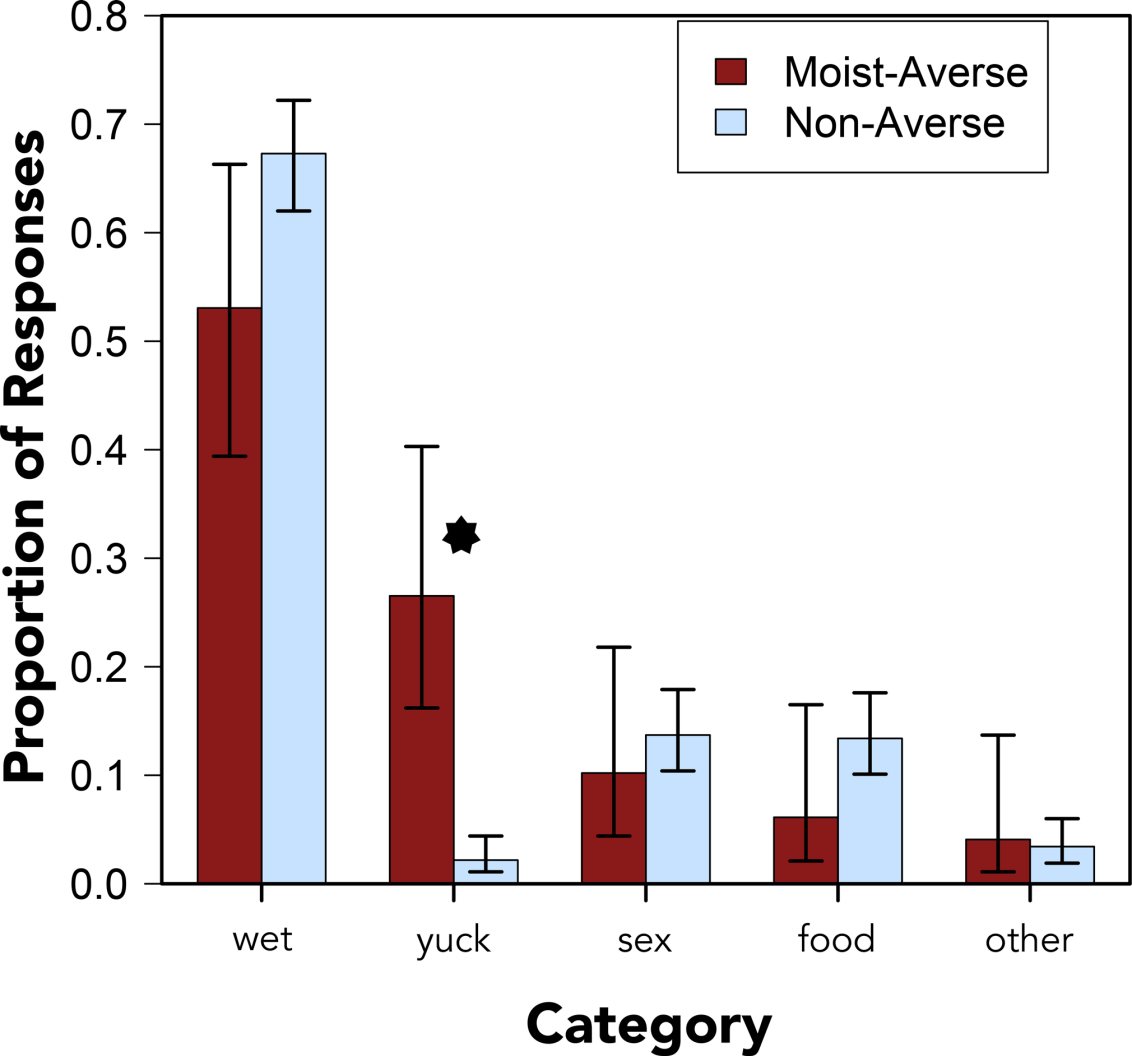
Lexical Associates. Proportions of lexical associates from moist-averse and non-averse participants by category. Error bars denote 95% confidence intervals. Asterisk indicates a statistically significant difference at the *p* < .001 level.

Free response data from Experiment 2 revealed that word aversion can be acute. In this experiment, participants were asked to write the first word that came to mind in response to each word in the set. Responses to “moist” were coded into one of five mutually exclusive categories–*wet*, *yuck*, *sex*, *food*, and *other*–and a chi-square test of independence revealed a significant difference in the kinds of words that averse and non-averse participants gave in response, *χ*^2^[df = 4, N = 370] = 50.200, *p* < .001, *V* = .737 (a similar result was obtained from an analysis in which the *other* category was removed, *χ*^2^[df = 3, N = 357] = 50.400, *p* < .001, *V* = .639; [34]). Moist-averse participants were noteworthy for their tendency to respond with a word like “yuck” or “eww”, *χ*^2^[df = 1, N = 370] = 44.648, *p* < .001, *V* = .347; they were marginally less likely to reply with a synonym for “moist” like “wet,” *χ*^2^[df = 1, N = 370] = 3.201, *p* = .074 (see Fig 1).

Finally, Experiment 3 revealed an influence of word aversion on memory. In this experiment, participants rated a set of 64 words and were then presented with a surprise recall task. The word “moist” was rated in the third of four blocks (fixed to position 38 of 64), yet was recalled by a surprisingly large number of participants (50.7%, *95%CI*: [.470, .544])–especially those who reported an aversion to the word (61.2%, *95%CI*: [.536, .695] compared to 47.9%, *95%CI*: [.438, .521]), χ^2^[1, N = 688] = 7.264, *p* = .007, *V* = .103 (see Table 4).

**Table 4:** Most and Least Frequently Recalled Words.

	Moist-Averse		Non-Averse	
Rank	Word	%	Word	%
**1**	Fuck	63.3	Fuck	62.8
**2**	Pussy	62.6	Pussy	61.9
**3**	Moist	61.9	Pus	56.8
**62**	Part	< 1	Number	1.6
**63**	Number	< 1	Part	1.3
**64**	Street	< 1	Street	< 1

Words recalled most and least often by moist-averse and non-averse participants.

Together data from these three experiments help to characterize what it means to be averse to a particular word. The phenomenon is characterized by a visceral response to the aversive word, which can be seen directly in subjective ratings of word *aversiveness*, and in the responses of participants in a free association task. In addition, people with an aversion to “moist” were significantly more likely to remember and report having encountered the word in a surprise recall task.

### How common is word aversion and who experiences it?

In Experiments 1–5, 20.5% (*n* = 82, *95%CI*: [.168, .247]), 15.1% (*n* = 57, *95%CI*: [.168, .247]), 18.9% (*n* = 108, *95%CI*: [.168, .247]), 13.2% (*n* = 49, *95%CI*: [.168, .247]), and 20.2% of participants (*n* = 139, *95%CI*: [.168, .247]) reported a categorical aversion to the word “moist.”

At least three methodological factors influenced this judgment: 1) when participants were asked the question, 2) whether the experiment involved a rating task, and 3) the dimensions along which ratings were made. First, participants were less likely to report an aversion to “moist” when they were asked to identify as categorically averse at the beginning of the study, before having rated the target items (Experiment 4) compared to the end, after they had rated the target items (Experiment 1), *χ*^2^[df = 1, N = 777] = 7.433, *p* = .006, *V* = .098. Second, participants were less likely to report an aversion to “moist” when they had engaged in a free response (Experiment 2) rather than a rating task (Experiment 1), *χ*^2^[df = 1, N = 770] = 6.664, *p* = .010, *V* = .093. Third, participants were more likely to find “moist” aversive after having rated it (and other target items) for a positive (24.9%, *95%CI*: [.206, .298]), rather than a negative (15.7%, *95%CI*: [.122, .198]), connotation (Experiment 3): *χ*^2^[df = 1, N = 688] = 8.572, *p* = .003, *V* = .112.

These results suggest that the rating task itself (indeed, nuances of the rating task) may have contributed to participants’ judgment about their own aversion to “moist,” and that word aversion may result, at least in part, from an explicit consideration of a word’s lexical properties. Further, for many people, a negative connotation of “moist” may be particularly salient or difficult to suppress, leading to a contrast effect when rating words for a positive connotation [33], as people were most likely to report a categorical aversion to “moist” after rating the word for a positive connotation (in Experiment 3).

To investigate who is likely to experience word aversion we analyzed demographic and personality variables. Table 5 shows the proportion of moist-averse participants by demographic and personality variable.

**Table 5:** Demographics of Word Aversion.

Variable	*n*	Exp1	Exp2	Exp3	Exp4	Exp5	*Overall*
Gender*							
Female	1484	0.220	0.176	0.253	0.208	0.220	0.222
Male	966	0.185	0.079	0.112	0.09	0.124	0.118
Age (*median* = 32; *range*: [18, 80])*							
Younger	1270	0.273	0.178	0.23	0.169	0.266	0.228
Older	1188	0.125	0.057	0.172	0.135	0.123	0.131
**Education (*median* = Associate’s Degree; *range*: [Less than HS, Doctorate])**							
Less	1306	0.219	0.132	0.174	0.124	0.198	0.173
More	1152	0.188	0.133	0.233	0.183	0.181	0.190
**Ideology (*median* = 41; *range*: [0, 100])**							
Liberal	1235	0.229	0.129	0.222	0.186	0.216	0.202
Conservative	1223	0.176	0.137	0.182	0.122	0.165	0.160
Blirtatiousness (*median* = 24; *range*: [8, 40])*							
Less	977	NA	0.157	0.224	0.175	0.22	0.203
More	980	NA	0.109	0.175	0.118	0.150	0.144
Disgust: Body (*median* = 5; *range*: [2, 6])*							
Less	859	NA	0.152	0.192	0.089	0.168	0.159
More	1175	NA	0.119	0.214	0.203	0.206	0.191
**Disgust: Sex (*median* = 5; *range*: [2, 6])**							
Less	551	NA	0.153	0.124	0.096	0.160	0.136
More	1479	NA	0.124	0.220	0.172	0.204	0.190
**Religiosity (*median* = 6; *range*: [2, 25])**							
Less	1211	NA	0.148	0.204	0.147	0.195	0.178
More	833	NA	0.100	0.199	0.170	0.182	0.175
**Extraversion (*median* = 5; *range*: [2, 10])**							
Less	1021	0.162	0.176	0.166	0.156	0.195	0.173
More	1437	0.221	0.109	0.240	0.148	0.185	0.186
**Agreeable (*median* = 6; *range*: [2, 10])**							
Less	1291	0.201	0.137	0.226	0.144	0.197	0.177
More	1167	0.231	0.114	0.194	0.174	0.186	0.186
**Conscientiousness (*median* = 7; *range*: [2, 10])**							
Less	1169	0.293	0.168	0.174	0.115	0.172	0.178
More	1289	0.176	0.116	0.256	0.164	0.221	0.184
Neuroticism (*median* = 6; *range*: [2, 10])*							
Less	1239	0.185	0.108	0.217	0.105	0.180	0.158
More	1219	0.246	0.183	0.194	0.250	0.195	0.204
**Openness (*median* = 7; *range*: [2, 10])**							
Less	1330	0.201	0.153	0.192	0.161	0.191	0.180
More	1125	0.213	0.087	0.213	0.131	0.189	0.182

Proportion of moist-averse participants by demographic and personality variable.

Asterisks indicate statistically significant differences at the *p* < .01 level.

To conduct statistical tests on the relationship between these individual difference measures and word aversion, we aggregated data across the four experiments that included all of the individual difference measures (Experiments 2–5, excluding those who declined to respond to any of these measures; *N* = 1,873 analyzed). We conducted separate tests of the relationships between the individual difference measures and moist-aversion, which revealed differences by gender, age, blirtatiousness, disgust toward bodily function, and neuroticism (see Table 5).

Due to the covariation between these measures, a logistic regression model with predictors for age, gender, sub-components of the disgust scale (e.g., disgust related to bodily function and disgust related to sex), religiosity, and the Big Five personality dimensions (openness, conscientiousness, extraversion, agreeableness, and neuroticism) was also fit to the data. To find the best fitting model, we utilized a stepwise model selection algorithm from the MASS library in *R* [35]. This algorithm takes a maximally parameterized model and tests alternatives that include subsets of predictor variables by comparing AIC values (by both pairing down from the maximally parameterized one and working up from the minimally parameterized one) in order to find the best fit for the data [36].

Table 6 shows the results of the best fitting model (*AIC* = 1602.8; *AIC* for the maximally parameterized model = 1615.2; *AIC* for a model without predictors = 1728.5), which suggests that the prototypical moist-averse person is a young, neurotic, female who is well-educated and somewhat disgusted by bodily function. This model is largely consistent with the results shown in Table 5, although the logistic regression model omitted the measure of blirtatiousness (which was significantly related to each of the variables that were included in the final model) and included participants’ educational background.

**Table 6:** Predicting Word Aversion by Individual Differences.

Estimate	*β*	*SE*	*p*
**Intercept**	-1.403	0.080	< .001
**Age**	-0.581	0.078	< .001
**Male**	-0.921	0.151	< .001
**Disgust: Body**	0.262	0.070	< .001
**Neuroticism**	0.157	0.067	0.020
**Education**	0.200	0.067	0.003

Results of best fitting logistic regression model, using the individual difference measures to predict moist-aversion.

The relationships between gender, age, and neuroticism to word aversion is consistent with prior work on disgust (e.g., [37–38]). Females, younger individuals, and people who express more neuroticism tend to be more sensitive to disgust. The influence of education seems more uniquely related to the phenomenon of word aversion.

The relationship between word aversion and disgust for bodily function, and not disgust for sex, suggests possible support for a specific semantic relatedness hypothesis–that aversion to “moist” may be grounded in associations to effluvia [39].

We tested for converging evidence for the hypothesis that word aversion is related to (specific) semantic associations of “moist” in the sections below by, for instance, investigating whether moist-averse participants were sensitive to the cluster of words related to bodily function (e.g., phlegm, puke, vomit), the cluster of words related to sex (e.g., fuck, horny, pussy), and to words with similar phonological properties to “moist” (e.g., foist, hoist, rejoiced).

### Is “moist” aversive because it sounds unpleasant or because it has unpleasant connotations?

In all five experiments, participants were asked to speculate on the source of word aversion in a free response task. Most people identified the semantic associations between “moist” and sex as the most likely culprit. However, the pattern differed as a function of whether participants identified as averse to the word. As shown in Table 7, moist-averse participants were more likely to identify phonological properties of the word as a contributor to word aversion than non-averse participants, suggesting possible support for a phonological cause of word aversion [4–8].

**Table 7:** Speculation on Cause of Word Aversion.

Experiment	Aversion	Sound	Connotation	*K*	*χ*^2^(3)	*χ*^2^(1)
**1** (N = 400)	Averse (*n =* 82)	40.2%	39.0%	.803	43.462	26.646
	Non-averse	11.3%	52.8%		*V* = .571	*V =* .315
**2** (N = 377)	Averse (*n =* 57)	38.6%	49.1%	.710	16.694	13.555
	Non-averse	15.6%	66.9%		*V* = .498	*V* = .231
**3** (N = 572)	Averse (*n =* 108)	51.9%	37.0%	.831	54.175	40.553
	Non-averse	19.0%	60.3%		*V* = .533	*V* = .296
**4** (N = 370)	Averse (*n =* 49)	51.0%	38.8%	.778	39.501	28.465
	Non-averse	15.0%	66.4%		*V* = .566	*V* = .305
**5** (N = 688)	Averse (*n =* 139)	47.5%	43.9%	.841	47.743	31.207
	Non-averse	19.9%	58.7%		*V* = .415	*V* = .293

Speculation on the cause of moist aversion in Experiments 1–5 grouped by self-reported moist-aversion. The “sound” column reflects the proportion of participants who identified the sound alone or the sound and connotation as aversive; the “connotation” column reflects the proportion of participants who identified the connotation alone as aversive. *K* refers to Cohen’s Kappa, a measure of inter-rater reliability. Two chi-square tests of independence are reported for each experiment: one in which all four categories of responses were taken into account, and one in which two categories were taken into account (sound, which collapsed over sound and connotation, and connotation alone; the “other” category was excluded for this second test). All *p*s < .001. Cramer’s *V* is reported as a measure of effect size.

If the sound of the word underlies peoples’ aversion to “moist” then one might expect moist-averse participants to rate words with similar phonological properties as aversive as well. However, if the semantic connotation of the word causes the aversion, then one would expect moist-averse participants to rate words with similar semantic properties as more aversive. Alternatively, it is also possible that there are no specific clusters of words to which moist-averse participants are selectively more sensitive (e.g., moist-averse participants may simply report high ratings of *aversiveness* to all words or only to “moist”)–a possibility that would support the fourth hypothesis: that word aversion is a fad or isolated phenomenon.

Two analyses of these data are presented. First, a two-way repeated measures ANOVA with predictors for moist aversion (yes or no) and word type (moist, semantically related, phonologically related, bodily function, sex, negative, positive) was used to model ratings of the *aversiveness* of the seven categories of words separately for data from Experiments 1, 4, and 5. Ratings of *aversiveness* were averaged by participant and word type, yielding, in Experiment 1, 2800 unique data points (i.e., for 7 word types for each of 400 participants).

A second set of analyses utilized mixed-effect linear regression models using the *lme4* package in *R* [40] and revealed consistent results. On this approach, ratings of individual words (as opposed to categories of words) were treated as the dependent variable. “Participant” and “item” (rated words) were included as random effects to simultaneously account for error variance associated with these factors [41–42]. Lexical categories (word type) and self-reported word aversion were treated as fixed effects (the independent variables). We report the results of comparisons between nested models, for which the amount of additional variance explained by including predictor variables (parameters) approximates a *χ*^*2*^ distribution with the number of added parameters as its degrees of freedom [43]. For brevity, we focus primarily on the results of the repeated measures ANVOA in the text.

**Fig 2:**
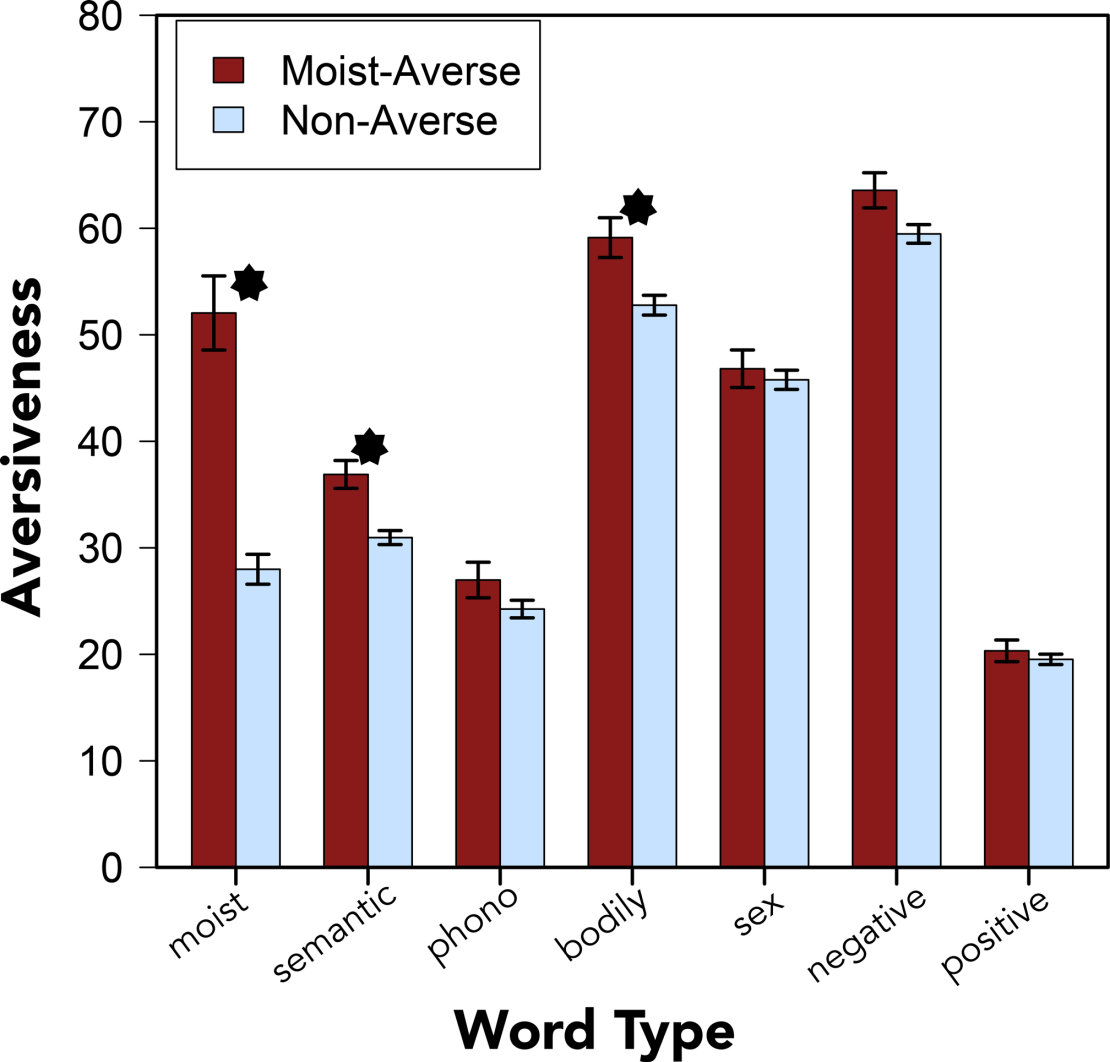
Aversiveness Ratings. Ratings of the *aversivness* of “moist” and words from six lexical categories from Experiment 1, grouped by participants who identified as moist-averse or non-averse. Error bars denote standard errors of the means. Asterisks indicate statistically significant differences at the *p* < .05 level.

In all three of these experiments, both main effects were statistically significant. The repeated measures ANOVA revealed that moist-averse participants rated the items as more *aversive* overall: Experiment 1, *F*[1, 398] = 17.336, *p* < .001, *η*^*2*^ = .042; Experiment 4, *F*[1, 375] = 49.087, *p* < .001, *η*^2^ = .116; Experiment 5, *F*[1, 570] = 40.471, *p* < .001, *η*^2^ = .066. And some categories of words were rated as more *aversive* than others, (e.g., the category of negatively valenced words was rated as more *aversive* than the category of positive words; see Fig 2): Experiment 1, *F*[6, 2388] = 247.181, *p* < .001, *η*^*2*^ = .367; Experiment 4, *F*[6, 2250] = 335.285, *p* < .001, *η*^2^ = .449; Experiment 5, *F*[6, 3420] = 413.522, *p* < .001, *η*^2^ = .409.

More revealing for the present investigation was an interaction between moist-aversion and word type: Experiment 1, *F*[6, 2388] = 11.360, *p* < .001, *η*^*2*^ = .017; Experiment 4: *F*[6, 2250] = 36.594, *p* < .001, *η*^2^ = .049; Experiment 5: *F*[6, 3420] = 26.785, *p* < .001, *η*^2^ = .027 (see Fig 2 and Table 8). The interaction between moist-aversion and word type was confirmed in the mixed-effect linear regression models for Experiment 1, *χ*^*2*^(6) = 59.741, *p* < .001, Experiment 4, *χ*^*2*^(6) = 204.340, *p* < .001, and Experiment 5, *χ*^*2*^(6) = 116.590, *p* < .001 (see right-most column of Table 8 for results of planned comparisons using the mixed model approach).

**Table 8:** Difference in Aversiveness Ratings by Word Type.

Type	Exp	*M*_*diff*_	*95%CI*		*t*	*d*	*χ*^*2*^
**Moist**	1	24.061	16.684	31.439	7.335***	0.854	NA
	4	47.535	39.452	55.618	14.964***	1.705	NA
	5	32.156	25.831	38.482	11.792***	1.131	NA
**Semantic related**	1	5.928	1.607	10.249	2.559*	0.315	6.52*
	4	14.500	9.037	19.963	5.451***	0.755	28.69***
	5	6.650	2.624	10.675	3.325**	0.352	10.97**
**Phono related**	1	2.727	-1.990	7.444	1.123	0.139	1.23
	4	4.792	-0.682	10.265	1.846	0.265	3.40
	5	5.132	1.185	9.079	2.655**	0.282	7.02**
**Bodily function**	1	6.346	0.668	12.025	2.204*	0.272	4.85*
	4	12.117	4.834	19.399	3.288**	0.467	10.72**
	5	5.850	1.056	10.643	2.295*	0.244	5.25*
**Sex**	1	1.040	-4.307	6.386	0.388	0.048	0.15
	4	8.010	1.181	14.839	2.159*	0.309	4.64*
	5	3.571	-1.224	8.366	1.487	0.159	2.21
**Negative**	1	4.092	-0.471	8.656	1.702	0.210	2.89
	4	6.840	1.234	12.445	2.192*	0.314	4.79*
	5	0.397	-3.923	4.716	0.176	0.019	2.21
**Positive**	1	0.802	-4.850	6.454	0.286	0.036	0.08
	4	-0.050	-5.001	4.902	0.021	-0.003	0.01
	5	4.897	-0.170	9.963	2.161*	0.230	4.66*

Differences between averse and non-averse participants’ ratings of the aversiveness of words from seven lexical categories for Experiments 1, 4, and 5. The *χ*^*2*^ statistic represents the additional variance explained by a mixed-effect linear regression model that included a test for an interaction between self-reported moist-aversion and word type. Asterisks indicate statistically significant differences at the *p < .05, **p < .01, ***p < .001 levels.

Planned comparisons showed that moist-averse participants reliably rated “moist,” words that were semantically related to “moist,” and words related to bodily function as more aversive than non-averse participants. This pattern of results supports the semantic relatedness account of word aversion–specifically, the hypothesis that moist-aversion is related to disgust toward bodily function (and not, e.g., sex).

Ratings of the four other categories of words (phonologically related, sexual, unrelated positive, and unrelated negative) yielded mixed results. In Experiment 4, moist-averse participants were more sensitive to words with a sexual connotation and to words that were more negative overall. These differences may be spurious (given the results of Experiment 1 and 5) or may have emerged as a result of the design of Experiment 4, in which participants were asked to identify as categorically moist-averse before the rating task. Participants who identified as moist-averse in Experiment 4 may have adopted a different strategy for rating the subsequent word list than participants in Experiment 1 or 5 (e.g., emphasizing the aversive nature of sexual words and words with a negative valence).

In Experiment 5, moist-averse participants were more sensitive to words that were phonologically similar to “moist” and to positive words. As with Experiment 4, these differences may be spurious. However, it may also be the case that moist-averse participants are, in fact, more sensitive to phonological properties of words like “moist” than non-averse participants. On this account, the additional power afforded by the larger sample size may have revealed a true (but fairly small and subtle) difference between moist-averse and non-averse participants. This account finds some support in the measure of effect size: the difference between averse and non-averse participants ratings of phonologically related words in Experiment 5 was similar to that of Experiment 4 (*d*s = .265 and .282 in Experiments 4 and 5, respectively; albeit larger than that of Experiment 1, *d* = .139).

**Fig 3:**
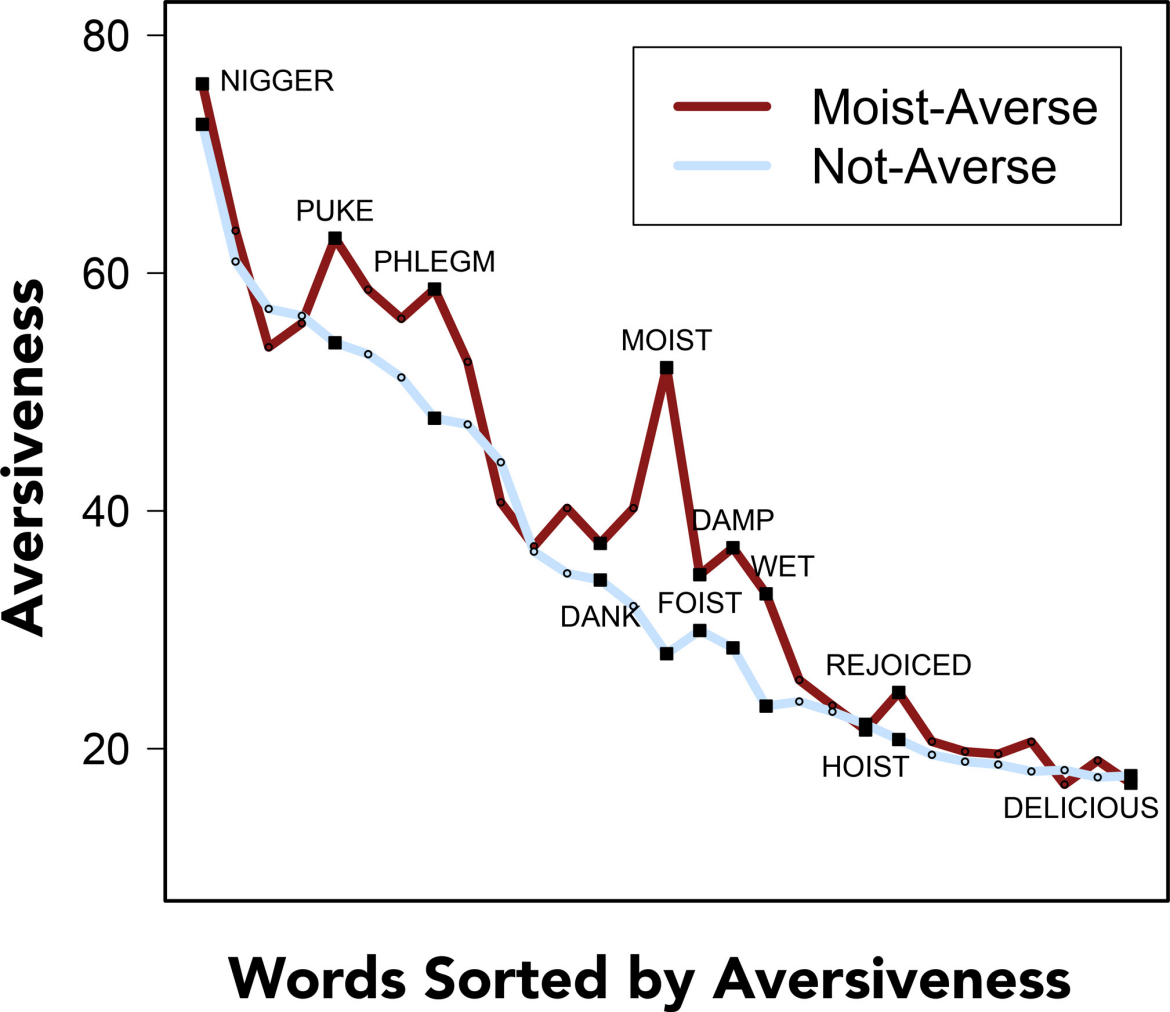
Rated words sorted from most to least aversive. Separate means of word *aversiveness* are presented for participants who reported an aversion to moist (dark red) and for participants who did not (light blue). A subset of words are identified in the plot as reference points.

Nevertheless, the clearest finding that emerges from these analyses is that moist-averse participants were reliably more sensitive to the aversive nature of words that were the most semantically related to “moist” (e.g., damp, wet) and words related to bodily function (e.g., puke and phlegm). Importantly, the word “moist” was localized at roughly the midpoint of the distribution of the *aversiveness* scale and moist-averse participants did not simply rate all of the words in the set as more *aversive* than non-averse participants (see Fig 3).

Contrary to the explicit speculation of many moist-averse participants, these results do not yield much support for accounts of word aversion that appeal to phonological features of the word (i.e., the sound symbolism or facial feedback hypotheses) or to accounts that link moist-aversion to disgust related to sex.

One reason that participants who identify as moist-averse may cite phonological features of the word as the source of their aversion is that their subjective experience of word aversion is so visceral and immediate that it seems to have been triggered by the sound of the word, even though this may not be the true cause of the aversion [24, 44–45].

Finally, additional support for the semantic related-ness account comes from a secondary analyses of data from Experiment 1, in which the semantic relatedness of words to “moist” was operationalized and quantified by corpus-based measure of contextual co-occurrence–latent semantic analysis (LSA) [46]. LSA has been shown to, among other things, reliably predict response times for semantically primed target words in a lexical decision task [47]. For context, the two words that were judged by LSA to be most similar to “moist” were “damp” (*cosine* = .64) and “wet” (*cosine* = .53); the two words that were judged by LSA to be least similar to LSA were “foist” (*cosine* = -.02) and “brave” (*cosine* = -.02).

This measure of semantic similarity reliably predicted mean differences in *aversivness* ratings between moist-averse and non-averse participants (excluding the word “moist” left difference scores from 28 words for this analysis): *β* = .518, *SE* = .168, *p* = .005, adjusted-*R*^*2*^ = .241 (*r*[26] = .518, *p* = .005). That is, differences in ratings of words’ *aversiveness* between moist-averse and non-averse participants could be explained in terms of their semantic relatedness to “moist,” as operationalized by contextual co-occurrence.

### Did people who found “moist” aversive also condemn incest?

The results so far suggest that “moist” is aversive because of its semantic connotation and may be grounded in a disgust elicited by bodily function. However, in Experiment 4, moist-averse participants also rated sexual words as more *aversive*, suggesting that feelings of disgust associated with sex may also contribute to word aversion.

To further explore the relationship between word-aversion and disgust, we asked participants in Experiment 5 to make a moral judgment about the acceptability of incest between siblings [23]. If moist-aversion is related to sex, one would expect moist-averse participants to find consensual incest to be less morally acceptable. An independent samples t-test revealed no difference between groups, *t*[570] = .941, *p* = .347, a result that, in concert with the ratings data and the analysis of individual measures, suggests that moist-aversion is more strongly related to a non-sexual aspect of disgust (i.e., to bodily function).

### What is the lexical profile of an aversive word?

Two methods were used to assess the lexical profile of “moist.” In Experiment 3, half of participants rated words for their positive connotation while the other half rated words for their negative connotation. One potentially distinguishing feature of the word “moist” (as well as many other words cited as aversive) is that it has both strongly positive and strongly negative connotations (e.g., associations with cake *and* armpits). These distinct senses of “moist” may lead to a dissonant experience of the word that imparts the aversion (e.g., the word may simultaneously call to mind cake and armpits). Evidence for such a possibility would be found if “moist” was rated as having both strongly positive and strongly negative connotations.

**Fig 4:**
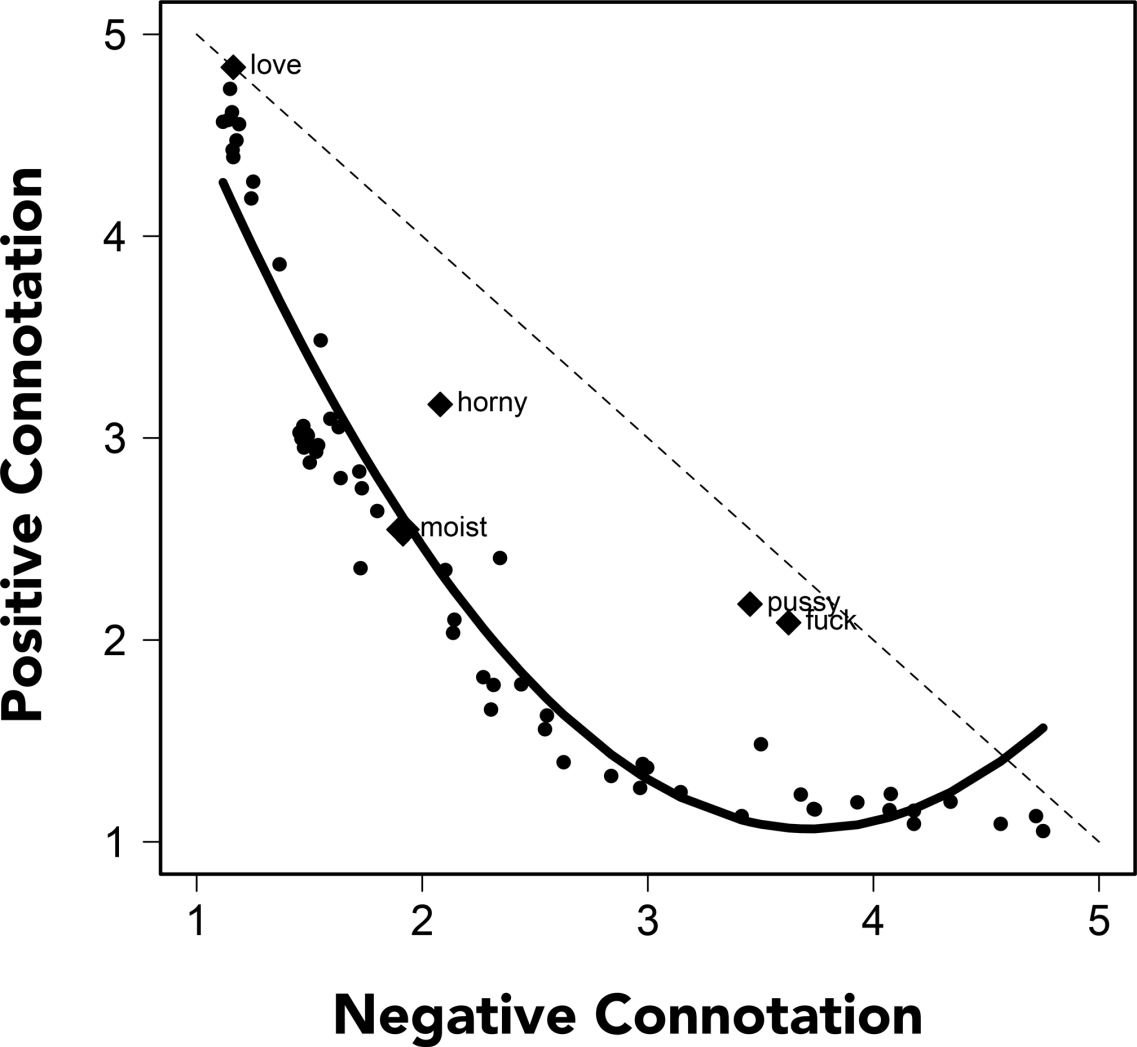
Positive and Negative Connotations. Relationship between ratings of the 64 words’ negative and positive connotations. The dotted line represents what would be expected if the ratings of the words’ negative connotations were perfectly anti-correlated with the words’ positive connotations. The solid line reflects predicted values from the regression line, which revealed linear and quadratic relationships between the ratings. Items that deviated from the general pattern and “moist” are identified with labels.

We found that, across the full set of items, ratings of positive and negative connotations were highly correlated, *r*[62] = -.863, *p* < .001. However, there was also a non-linear relationship between the positive and negative ratings of the target words. A regression model revealed significant linear, *β* = -6.852, *SE* = .316, *p* < .001, and quadratic, *β* = 3.158, *SE* = .316, *p* < .001, relationships between the positive and negative ratings of the words (adjusted-*R*^2^ = .900). According to a Kolmogorov-Smirnov Test, the two sets of ratings data were similarly distributed, *D* = .141, *p* = .552; however, as shown in Fig 4, the relationships between the positive and negative words differed as a function of how positive/negative the words were. The solid line in Fig 4 depicts this relationship as it was characterized by the regression model (i.e., with a combination of linear and quadratic functions). Words that were rated has having only a slightly negative connotation (roughly less than 2.5) showed a strong linear correlation between ratings of their positive and negative connotations, *r*[38] = -.903, *p* < .001; words that were rated has having a more negative connotation (greater than 2.5) were judged as having a fairly low positive connotation. The positive and negative connotation ratings for these words was weaker, *r*[22] = -.449, *p* = .028, and showed less variability along the positive dimension (*s*^*2*^ = .059 compared to *s*^*2*^ = .628, *F*[1, 62] = 89.050, *p* < .001, *η*^2^ = .590). The words “pussy” and “fuck” were notable exceptions to this pattern: possibly because these words can be used both pejoratively and to describe positive sexual experiences.

“Moist” was rated close to the midpoint of the positive (*M* = 1.915, *95%CI*: [1.867, 1.962]; *median* for all words = 2.381) and negative scales (*M* = 2.546, *95%CI*: [2.496, 2.596]; *median* for all words = 2.091). Unlike “pussy” and “fuck,” and contrary to our prediction, it was not rated as having both unusually strong positive and negative connotations.

A second way in which we sought to characterize the lexical profile of aversive words was by quantifying “moist” along six target dimensions (Experiments 1, 4, and 5)–*personal use*, *familiarity*, *aversiveness*, *valence*, *arousal*, and *imagery–*a paradigm that has been used to study the lexical profile of taboo and emotionally valenced words [25]. In prior work, taboo and disgusting words were found to be associated with a negative *valence* and a large difference between, on the one hand, *familiarity*, and on the other hand, *personal use* and *offensiveness*: people were highly familiar with these words but did not use them and found them offensive. Neither taboo nor disgusting words were noteworthy for their *imageability* or *arousal*, relative to other positive and negative words.

**Fig 5:**
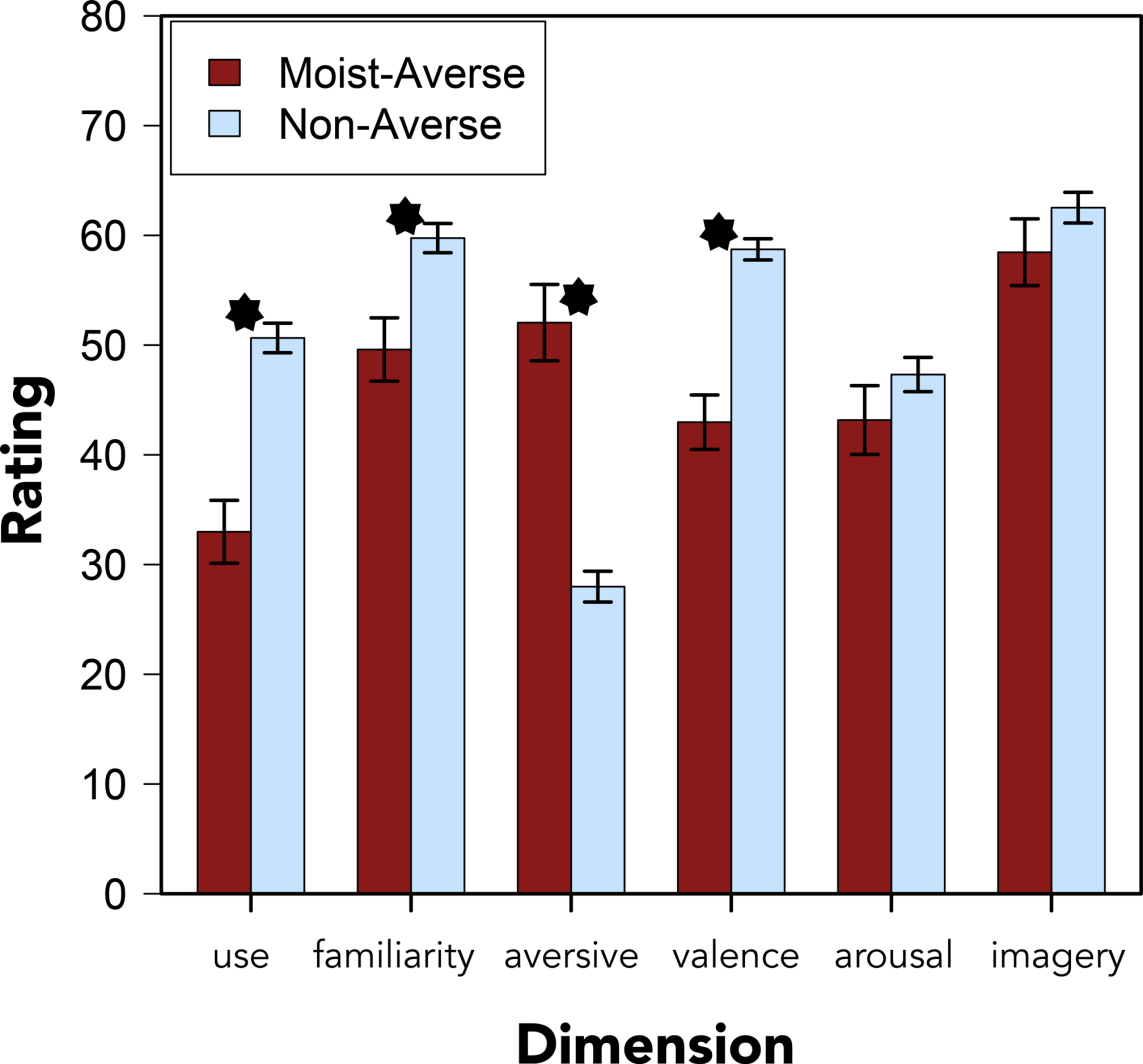
Moist Ratings by Aversion. Ratings of “moist” along six dimensions grouped by participants who identified as moist-averse or non-averse. Error bars denote standard errors of the means. Asterisks indicate statistically significant differences at the *p* < .001 level.

For participants who reported an aversion to “moist,” the pattern of ratings data for “moist” showed some similarity to this profile: “moist,” for averse participants, was notable for its *aversiveness*, *valence*, and *personal use*, rather than *imagery* or *arousal*. However, unlike what was found for taboo words, moist-averse participants also reported less *familiarity* for “moist” compared to non-averse participants (see Table 9 and Fig 5). One explanation for this difference is that averse participants may have a more negative sense of “moist” in mind when making this rating, thereby making the word seem less familiar.

**Table 9:** Differences in Rated Dimensions of Moist.

Dimension	*M*_diff_	95%CI		*t*[398]	*p*	Cohen’s *d*
**Personal use**	-17.663	-23.61	-11.72	5.840	< .001	.695
**Familiarity**	-10.154	-16.06	-4.25	3.379	< .001	.413
**Aversiveness**	24.061	17.61	30.51	7.335	< .001	.854
**Valence**	-15.751	-20.23	-11.27	6.909	< .001	.810
**Arousal**	4.147	-2.66	10.95	1.199	.231	.148
**Imagery**	4.062	-2.15	10.27	1.287	.199	.159

Differences between averse and non-averse participants’ ratings of “moist” (averse minus non-averse) along the six target dimensions.

### Does recent experience modulate the *aversiveness* of “moist”?

Finally, in Experiment 5, we tested whether showing participants a video that was designed to highlight the aversive nature of “moist” would change the profile of the word and make it seem more aversive. Participants in this experiment either watched a video produced by *People Magazine* [1], which was designed to highlight the “cringeworthy” nature of the word, a control video, in which “moist” was used to describe a delicious cake, or no video, before rating the word.

Six separate one-way (by condition: *People*, cake, no video) ANOVAs were fit to analyze ratings of *personal use*, *familiarity*, *aversiveness*, *valence*, *arousal*, and *imagery*. The analysis revealed that the videos significantly impacted ratings of *personal use*, *F*[2, 569] = 4.573, *p* = .011, *η*^*2*^ = .016, *aversiveness*, *F*[2, 569] = 4.023, *p* = .018, *η*^*2*^ = .014, *valence*, *F*[2, 569] = 19.140, *p* < .001, *η*^*2*^ = .063, and *imagery*, *F*[2, 569] = 3.129, *p* = .045, *η*^*2*^ = .011, but not *familiarity*, *F*[2, 569] = .467, *p* = .627, *η*^*2*^ = .002, or *arousal*, *F*[2, 569] = .800, *p* = .450, *η*^*2*^ = .003.

**Fig 6:**
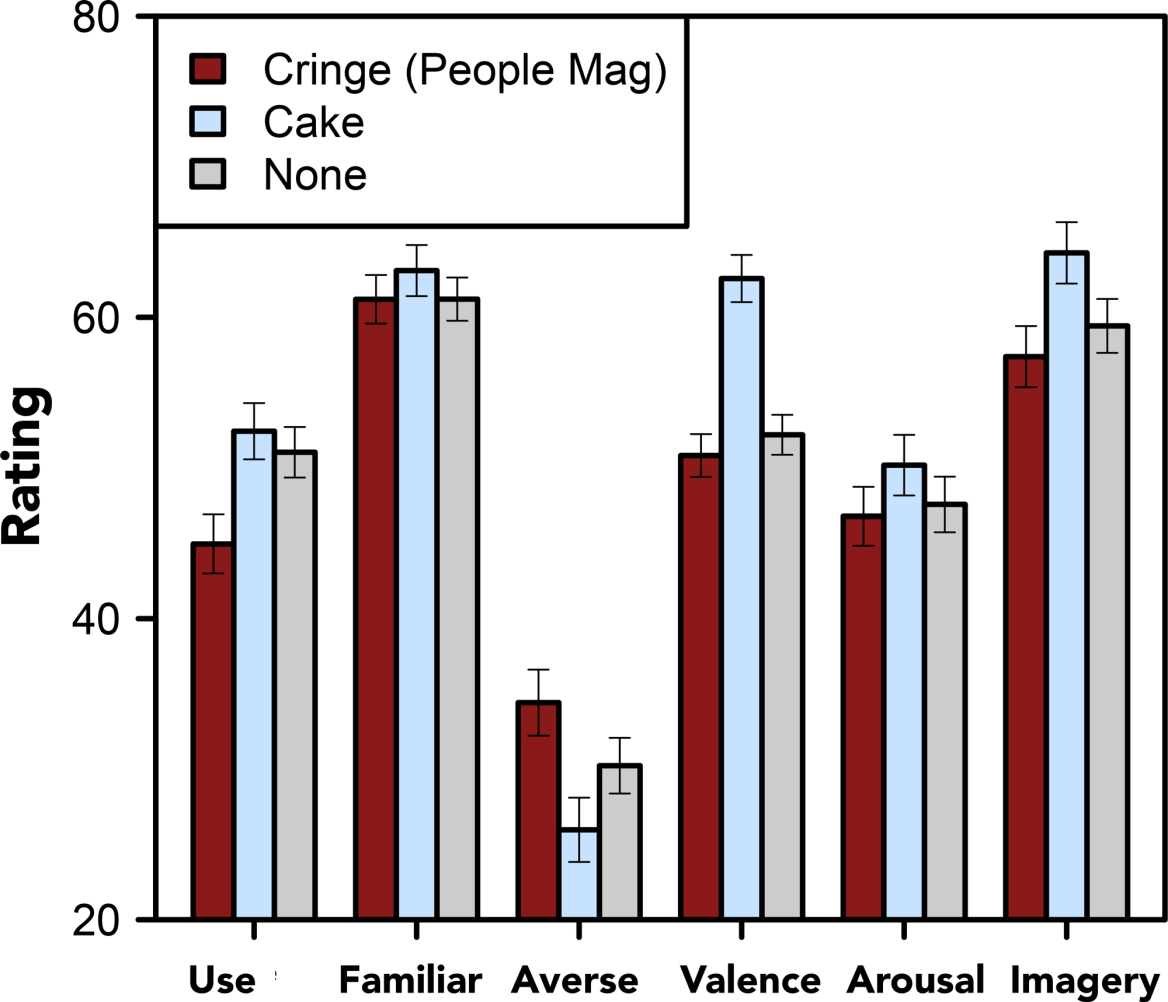
Moist Ratings by Condition. Ratings of “moist” along six dimensions grouped by condition: immediately before rating “moist” participants either watched a video designed to make “moist” seem cringeworthy, a video designed to prime a positive culinary sense of the word, or no video. Error bars denote standard errors of the means.

As shown in Fig 6, exposure to the video designed to make “moist” seem cringeworthy yielded lower ratings of *personal use*, *t*[570] = 2.977, *p* = .003, *d* = .264, and higher ratings of *aversivness*, *t*[570] = 2.420, *p* = .016, *d* = .241, compared to the no video condition. Exposure to the video in which “moist” was used to describe a cake, on the other hand, yielded lower ratings of *aversiveness*, *t*[570] = 2.423, *p* = .016, *d* = .245, a more positive *valence*, *t*[570] = 6.152, *p* < .001, *d* = .434, and more *imagery*, *t*[570] = 2.386, *p* = .017, *d* = .229, compared to the no video condition.

That is, watching the video that was designed to make “moist” seem cringeworthy not only made the word more *aversive*, it shifted the profile of the word to be more consistent with that of taboo, disgusting, and aversive words in general. For instance, after seeing the cringe-inducing video participants reported that they used the word less often.

## Discussion

The results of five experiments represent a novel exploratory effort to better understand the psychological underpinnings of word aversion. We have identified several methods for characterizing the subjective experience of word aversion and some of the implications for word aversion on behavior. For instance, we found that the difference in the rated *aversiveness* of “moist” between averse and non-averse participants was comparable to the difference in rated *aversivess* between “nigger” (arguably the most taboo word in American English) and “phlegm” (a relatively benign word in American English; situated near the midpoint of the scale). We also found, in a free association task, that moist-averse participants had strong associations between “moist” and visceral expressions of disgust like “eww” and “yuck.” We also found that people who reported an aversion to “moist” were about 25% more likely to remember having encountered the word in a surprise recall task.

Building on prior work related to emotionally valenced language like taboo words [25], we were also able to describe a lexical profile of aversive words. People who reported an aversion to “moist” reported less *familiarity* with and *personal use* of the word; they also considered the word to be more negatively *valenced*. Like taboo words, aversive words were not rated as more *arousing* or for the amount of *imagery* they brought to mind. Some of these patterns emerged in Experiment 5 as well, in which a group of participants were exposed to a video designed to make “moist” seem cringeworthy. In this study, the video not only made the word seem more aversive, it also made people think they used the word less often.

One hypothesis that was not supported by the data was that aversive words are unique for their strong associations to both positive and negative words. That is, one possibility we considered was that “moist” is aversive because of simultaneous positive (e.g., to eating cake) and negative associations (e.g., to armpits). Support for this prediction would have been found in disparate ratings of the word’s positive and negative connotations. However, the results of the rating task did not support this possibility.

Nevertheless, this experiment did reveal an interesting effect of the framing manipulation. People who rated “moist” for positive connotations were significantly more likely to identify as categorically averse to the word than people who rated “moist” for negative connotations. This, as well as the tendency for participants to be more likely to report an aversion to “moist” following rating tasks (Experiments 1, 3, and 5, compared to Experiments 2 and 4), suggests that explicit consideration of a word’s lexical features may contribute to word aversion. Specifically, thinking of the positive connotations of the word may be particularly likely to elicit categorical word aversion. This latter finding may be the result of a contrast effect [33]: people find negative things to be more negative when they’re placed in a positive context.

An additional contribution of the present studies is a systematic attempt to quantify the prevalence of word aversion and to identify characteristics of individuals who are likely to experience word aversion. Results of the experiments suggest that as much as 20% of the American English speaking population may be averse to “moist” and that this aversion is related to age, gender, neuroticism, education, and a particular kind of disgust to bodily functions (rather than, e.g., sex–and possibly a more general association to effluvia [39]).

The conclusion that moist-aversion is related to a disgust to bodily function was supported by analyses of the individual difference measures and the selective clusters of words that moist-averse participants tended to find aversive: words that were the most semantically related to “moist” like “wet” and “damp” as well as words that were related to bodily function like “phlegm” and “puke”; but not words related to sex like “horny” and “fuck” or words that had similar phonological properties to “moist” like “foist” or “hoist.” These findings contradict the explicit speculation of participants who reported an aversion to moist, who often cited phonological properties of the word as the source of their aversion, and the explicit speculation of the majority of participants, who tended to cite the word’s sexual connotation as a potential source of aversion.

The present work finds minimal support for the view that moist-aversion relates to the phonological features of “moist.” People who are averse to the word sometimes equate hearing “moist” to fingernails scratching a chalkboard. Experimental investigations of why people find fingernail screeches unpleasant suggest a similar tension between participants’ subjective experience and the underlying cause of the unpleasant reaction, as people are more likely to find fingernail scratches aversive when they know they are hearing fingernail scratches *per se* (i.e., the same sound out of context is perceived as less unpleasant) [48–51].

However, it is worth noting that the word “moist” seems to resonate as aversive more broadly in the general population of American English speakers compared to words that have very similar semantic properties (e.g., damp, wet, sticky). To our knowledge there are not Facebook pages, feature articles in newspapers, or plotlines of popular TV shows devoted to exposing the aversive nature of these words–as there are for “moist”–which seem more phonologically palatable. In future research, tools like EMG may help to shed further light on the facial feedback hypothesis and the phonological component of word aversion [52].

In addition, we found that moist-averse participants showed similar patterns of acceptance (or condemnation) of consensual incest as non-averse participants, which further suggests that the source of moist-aversion is related to disgust toward bodily function rather than sex. However, word aversion does appear to have certain properties in common with this kind of moral condemnation, in that both seem to be linked to a visceral experience of disgust, which is difficult to identify though introspection (i.e., a moral or aversion dumbfounding). This suggests that disgust responses may often go unnoticed when influencing a person’s judgment or behavior.

Identifying the relationship between word aversion and disgust also supports theoretical accounts of disgust that incorporate cultural factors [20] and not just biological ones [53]. Anecdotal evidence suggests that moist-aversion may be, in part, a viral phenomenon: the word has become contaminated through social and traditional media (e.g., a Facebook group titled “I HATE the word MOIST” has thousands of members; there have been entire plot lines of popular American TV shows like *How I Met Your Mother* and *The New Girl* devoted to the comic consequences of word aversion; and feature articles have been written about the topic in *Slate Magazine*, *The New Yorker*, *The Huffington Post* as well as other popular news outlets (e.g., [54–57]). In future work we plan to explore the cultural contribution of word aversion by investigating different populations. For instance, is word aversion prevalent among non-native English speakers, speakers of English from other countries (e.g., the UK, Australia, South Africa, the Caribbean), and people who communicate primarily via sign language?

Finally, an important aim of future work will be to further explore the prevalence and cause of word aversion with additional lexical items. In recent years, the word “moist” has been consistently cited as the “ugliest” or most “cringeworthy” word in American English [54–57]. However, a number words show up repeatedly on these lists. Some, predictably, relate directly to bodily function (e.g., among the top-10 ugliest words in a recent poll were “vomit,” “puke,” “phlegm,” “snot,” “damp,” and “mucus”; [57]). Others seem more noteworthy for their phonological properties (e.g., “slacks,” “crevice,” “luggage,” “pugilist,” “hardscrabble”; [56]). The methods presented here may serve as a model for investigating word aversion more broadly and for identifying additional relationships between aversive language and other psychological phenomena.

## Supporting Information

S1 Fig Mean Recall. Mean recall for “moist” and words from six lexical categories grouped by participants who identified as moist-averse or non-averse in Experiment 3.
(TIF)

S2 Fig Percentage Recall by Position. Recall by position of the word in the list in secondary Experiment 3.
(TIF)

S1 Text Supporting Information. Supplemental analyses and description of secondary Experiment 3.
(DOCX)
