# Supplementary figures and images for: A Moist Crevice for Word Aversion: In Semantics Not Sounds
Source: PLoS One. 2016 Apr 27;11(4):e0153686. doi: 10.1371/journal.pone.0153686 (PMC4847929; doi:10.1371/journal.pone.0153686)

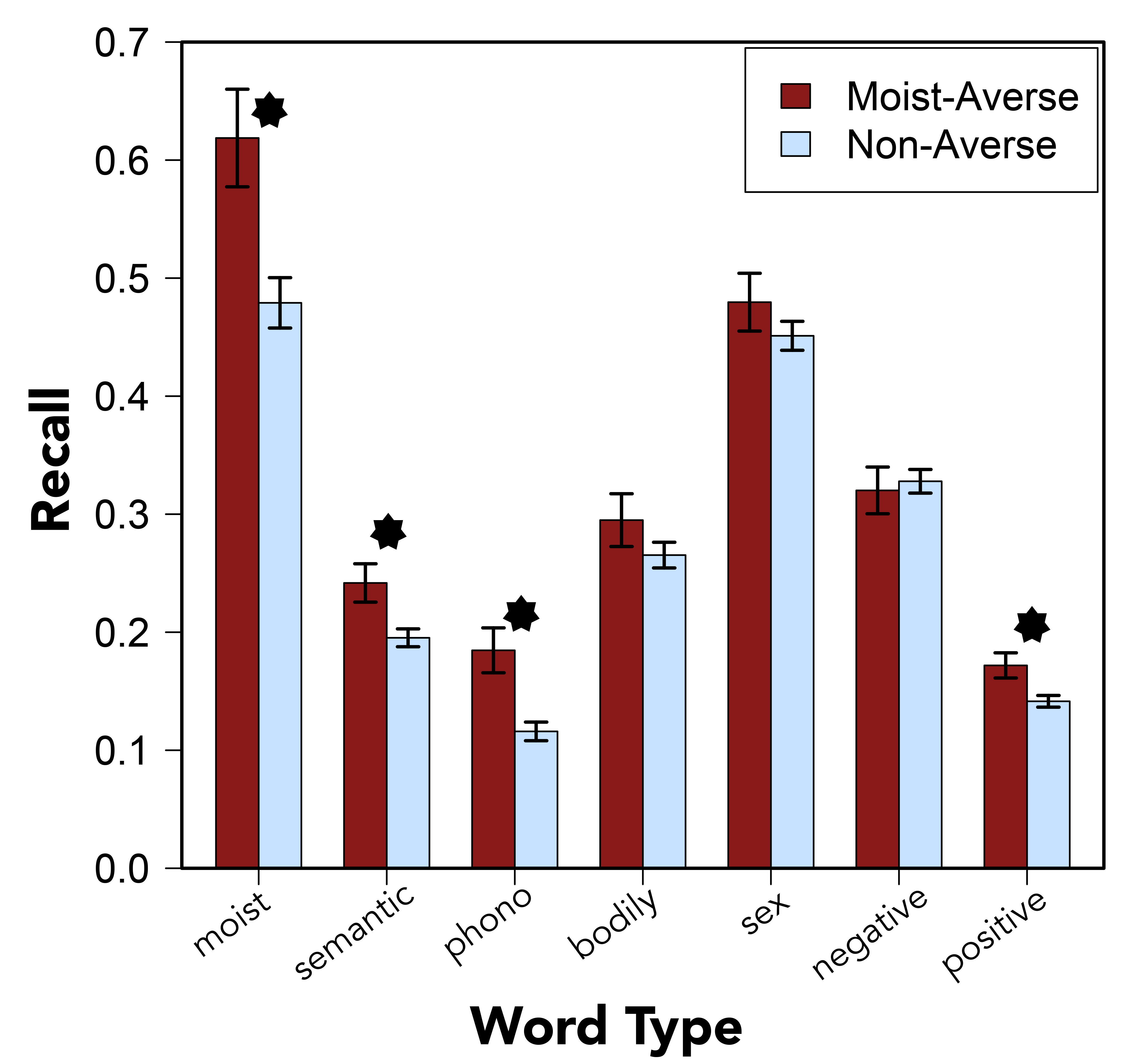

Supplement: S1 Fig — Mean recall for “moist” and words from six lexical categories grouped by participants who identified as moist-averse or non-averse in Experiment 3. (TIF) [file pone.0153686.s001.tif]

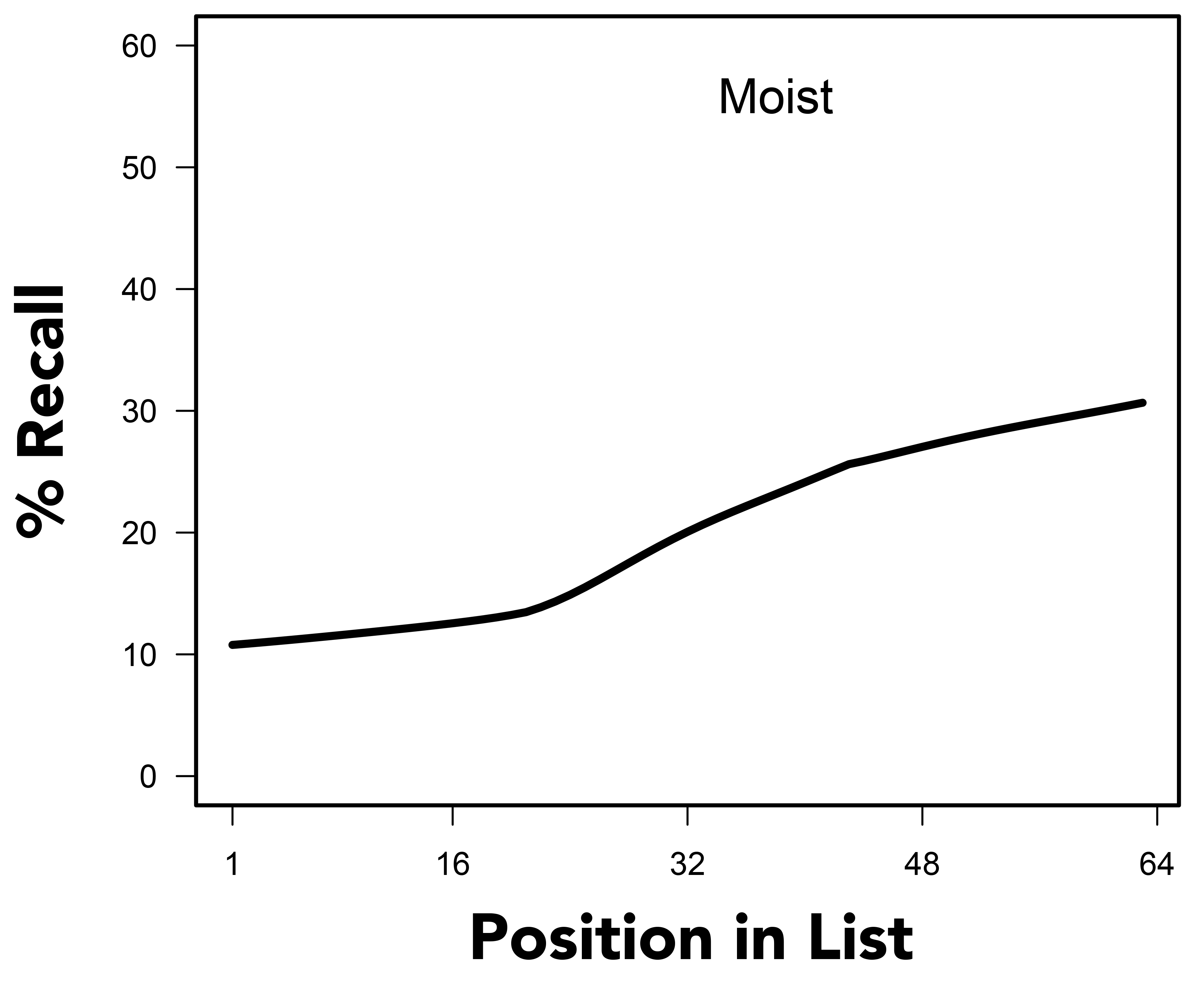

Supplement: S2 Fig — Recall by position of the word in the list in secondary Experiment 3. (TIF) [file pone.0153686.s002.tif]
