# Supplementary material for: A Moist Crevice for Word Aversion: In Semantics Not Sounds
Source: PLoS One. 2016 Apr 27;11(4):e0153686. doi: 10.1371/journal.pone.0153686 (PMC4847929; doi:10.1371/journal.pone.0153686)
Supplement: S1 Text — Supplemental analyses and description of secondary Experiment 3. (DOCX) [file pone.0153686.s003.docx]

**A Moist Crevice for Word Aversion:**

**In Semantics not Sounds**

In this supplement, additional analyses and figures are presented that may be of interest to some readers but were not directly related to the primary hypotheses. Note that the full datasets are available through the Open Science Framework: osf.io/3jwd4.

# Context Manipulation

As reported in the primary results section, ratings of the aversiveness of “moist” from Experiment 1 differed as a function of the context manipulation (i.e., whether participants had rated positive or negative words that were related or unrelated to “moist” immediately before rating “moist”). This was also the case in Experiment 4, *F*[3, 373] = 3.271, *p* = .021, *η*^2^ = .026: “moist” was considered the most aversive when it was primed to have a sexual connotation and least aversive when it followed unrelated negative words.

There was also an effect of the context manipulation in the free association task (Experiment 2), χ^2^[df=3,N=370] = 18.647, *p* < .001, *V* = .389. People were more likely to generate a word related to sex when “moist” followed sexual words (18% compared to 12% in the other conditions), χ^2^[df=3, N=370] = 7.905, *p* = .048, *V* = .253. People were more likely to generate a word related to food when “moist” followed “cake” (33% compared to 5% in the other conditions), *χ*^2^[df=3,N=370] = 48.560, *p* < .001, *V* = .627.

# Surprise Recall

In addition to analyses reported in the main paper related to the surprise recall task (Experiment 3), we tested whether moist-averse participants were more likely to recall words from the other categories of lexical items. We found that moist-averse participants were more likely to recall semantically related words, χ^2^[1, N = 688] = 5.846, *p* = .016, *V* = .092, words that were phonologically related to “moist”, χ^2^[1, N = 688] = 10.973, *p* < .001, *V* = .126, and positively valenced words, χ^2^[1, N = 688] = 5.632, *p* = .018, *V* = .090. There was no difference in rates of recall for sexual words, words related to bodily function, or negatively valenced words, χ^2^[1, N = 688]s < 1.5, *p*s > .25 (see Fig S1).

**S1 Fig. Mean Recall.** Mean recall for “moist” and words from six lexical categories grouped by participants who identified as moist-averse or non-averse. Error bars denote standard errors of the means. Asterisks indicate statistically significant differences at the *p* < .05 level.

# Alternate Experiment 3 (Not presented in main paper)

Experiment 3A was replaced in the main paper by Experiment 3. There are two main differences between Experiment 3A and Experiment 3:

1. In Experiment 3A, due to a coding error, participants were not asked whether they personally found the word “moist” aversive. This is why we chose to rerun the experiment.
2. In Experiment 3A, participants were asked to rate the target words for their “pleasantness” or “unpleasantness” rather than for their positive or negative connotation. We changed the labels of the scales for Experiment 3 because ratings of pleasantness and unpleasantness were highly correlated, *r*[62] = -.998, *p* < .001 (see below), suggesting that these labels did not have the intended effect on participants behavior.

The methods for Experiment 3A were otherwise identical to those of Experiment 3.

## Results

Overall, 55.9% of participants recalled that they had rated the word “moist” – it was the third most recalled item (after “pussy” and “fuck”, which were remembered 69% and 68% of the time, respectively). Neither the framing manipulation, χ^2^[3, N = 718] = .961, *p* = .811, nor the context manipulation, χ^2^[1, N = 718] = .004, *p* = .947, affected participants’ likelihood of recalling the word “moist.” There was no interaction between these factors, χ^2^[3, N = 718] = 2.015, *p* = .569 (See Table S1).

**Table S1. Moist Recall by Condition.**

|  | **Sex** | **Negative** | **Positive** | **Food** |
| --- | --- | --- | --- | --- |
| **Pleasantness** | 53% | 52% | 57% | 63% |
| **Unpleasantness** | 56% | 56% | 56% | 54% |

Percentage of participants who recalled “moist” by framing and context manipulations.

Fig S2 shows participants’ likelihood of recalling an item, given the item’s position on the survey. In general, the later the word in the list, the more likely it was recalled, *r*[62] = .732, *p* < .001. However, the word “moist” represented a notable exception to this pattern. Despite being presented in the middle of the survey, people – not just moist-averse participants – were surprisingly likely to recall having rated it.

**S2 Fig. Percentage Recall by Position.** Recall by position of the word in the list. “Moist” was always presented at position 38 and was recalled 56% of the time. Lowess smoothing was applied to the mean recall line to facilitate presentation of the trend.

Overall, ratings of pleasantness were highly correlated with ratings of unpleasantness, *r*[62] = -.998, *p* < .001. “Moist” was close to the midpoint along both dimensions: pleasantness, *M* = 3.722, *sd* = 2.407 (all words: *M* = 3.70, *sd* = 1.369) and unpleasantness, *M* = 4.196, *sd* = 1.251 (all words: *M* = 4.283, *sd* = 1.303). That is, the word “moist” was not rated as particularly pleasant (or unpleasant). Instead, the word was situated close to the midpoint and in roughly the same location on the dimensions of pleasantness and unpleasantness relative to the other lexical items. Thus, we find no support for the hypothesis that “moist” has conflicting associations – some highly positive, others highly negative – which gives rise to the aversion.
